# The correlationship between the metabolizable energy content, chemical composition and color score in different sources of corn DDGS

**DOI:** 10.1186/2049-1891-4-38

**Published:** 2013-09-25

**Authors:** Yong-Z Jie, Jian-Y Zhang, Li-H Zhao, Qiu-G Ma, Cheng Ji

**Affiliations:** 1 State Key Laboratory of Animal Nutrition, College of Animal Science and Technology, China Agricultural University, Beijing 100193, People's Republic of China

**Keywords:** Metabolizable energy, Distillers dried grains with solubles, Rooster, Predictive equation

## Abstract

**Background:**

This study was conducted to evaluate the apparent metabolizable energy (AME) and true metabolizable energy (TME) contents in 30 sources of corn distillers dried grains with solubles (DDGS) in adult roosters, and establish the prediction equations to estimate the AME and TME value based on its chemical composition and color score.

**Methods:**

Twenty-eight sources of corn DDGS made from several processing plants in 11 provinces of China and others imported from the United States. DDGS were analyzed for their metabolizable energy (ME) contents, measured for color score and chemical composition (crude protein, crude fat, ash, neutral detergent fiber, acid detergent fiber), to predict the equation of ME in DDGS. A precision-fed rooster assay was used, each DDGS sample was tube fed (50 g) to adult roosters. The experiment was conducted as a randomized incomplete block design with 3 periods. Ninety-five adult roosters were used in each period, with 90 being fed the DDGS samples and 5 being fasted to estimate basal endogenous energy losses.

**Results:**

Results showed that the AME ranged from 5.93 to 12.19 MJ/kg, TME ranged from 7.28 to 13.54 MJ/kg. Correlations were found between ME and ash content (-0.64, *P* < 0.01) and between ME and yellowness score (0.39, *P* < 0.05) of the DDGS samples. Furthermore, the best-fit regression equation for AME content of DDGS based on chemical composition and color score was AME = 6.57111 + 0.51475 GE - 0.10003 NDF + 0.13380 ADF + 0.07057 fat - 0.57029 ash - 0.02437 L (R^2^ = 0.70). The best-fit regression equation for TME content of DDGS was TME = 7.92283 + 0.51475 GE - 0.10003 NDF + 0.13380 ADF + 0.07057 fat - 0.57029 ash - 0.02437 L (R^2^ = 0.70).

**Conclusions:**

This experiment suggested that measuring the chemical composition and color score of a corn DDGS sample may provide a quality parameter for identifying corn DDGS sources energy digestibility and metabolizable energy content.

## Background

Distillers dried grains with solubles (DDGS) is a co-product from the ethanol industry, which is the residual component of the grain kernel after the starch has been fermented. In the United States the productions and supply of DDGS is increasing annually. Currently, the increased production of DDGS has been widely used in ruminant and swine feeding. In 2011, the majority (80%) of DDGS was fed to ruminants. The swine industry used nearly 10% of DDGS, whereas the poultry industry used around 9% of total DDGS [1]. However, previous researches had demonstrated that DDGS could be incorporated into laying hen diets at levels up to 15% to maintain egg production and had no negative effect [2-5]. In 2009, China was the second largest corn producer in the world. Besides China had become the third largest ethanol fuel producer after Brazil and the U.S. [6]. In 2006–2007, China DDGS production was 3.54 million tons and the productions was increasing annually [7], but only about 0.6 million tons DDGS from the ethanol industry and approximately 3 million tons mainly from drinking wine industry [8]. In 2010, China imported about 3.02 million tons DDGS from The U.S. and China had become the largest import country of America’s DDGS [9].

DDGS is not a completely homogenous ingredient. Differences in processing procedures and grain source may lead to large variations in the nutritional value of DDGS [10,11]. Furthermore, variable DDGS composition and instable quality may ultimately limit its use in poultry diets. Research had demonstrated the quality of DDGS could be evaluated based on color and chemical composition, because darker and high fiber content of DDGS results in lower TME [12-14] and amino acid digestibility [12,15-17]. However, there is little information about the correlation between ME content and chemical composition of DDGS from China.

Some previous studies [17,18] have indicated that the TME content of DDGS varied from 2,490 to 3,190 kcal/kg. Based on the instable quality of DDGS, it is important for marketers and buyers to develop a rapid method of evaluating metabolizable energy of DDGS. The objective of this study was to measure the AME and TME content in 30 sources of corn DDGS in adult roosters, and establish the prediction equations to estimate the metabolizable energy value based on their chemical composition and color score.

## Methods

### Samples of corn DDGS

Thirty sources of corn DDGS (the wheat DDGS, soghum DDGS and blend DDGS were not included in the present study) from ethanol plants in 11 provinces of China and the United States were used in this experiment (Table 1). The content of AME and TME in each source of DDGS was measured using roosters. All DDGS samples were ground using a Wiley Mill (model 8, Xingshi Scientific, BJ) equipped with a 2-mm screen, before being measured color score and fed roosters. The DDGS sources were analyzed for AME, TME and the degree of lightness (L*), redness (a*), and yellowness (b*) was measured using the Hunterlab colorimeter (model sc-80c, Kang Guang Photo Imaging China Inc., SPTCY 17017, BJ). Reported color score was the mean of 6 measurements, with the sample being mixed between each determination. Low values for L*, a* and b* indicated a dark color and lower degrees of redness and yellowness, whereas higher scores indicated a light color and greater degrees of redness and yellowness, respectively.

**Table 1:** The origin of the thirty kinds of distillers dried grains with solubles

**No.**	**The origin of DDGS**	
	**Company name**	**Place of production**
1	Huaxing biological Chemical Co., Ltd.	Mengzhou city, Henan Province
2	Tianguan Biological Chemical Co., Ltd.	Linying city, Henan Province
3	Zhengkui Co., Ltd.	Xinxiang city, Henan Province
4	Nanyang Tianguan Group Co., Ltd.	Nanyang city, Henan Province
5	Huaxing Alcohol Co., Ltd.	Nanyang city, Henan Province
6	Heyang Alcohol Industrial Co., Ltd.	Jiaozuo city, Henan Province
7	Mengzhou Luyuan lees processing plant	Mengzhou city, Henan Province
8	Yiwang Zhongyuan biotechnology Co., Ltd.	Xinzheng city, Henan Province
9	Fuel alcohol Co., Ltd. (2011)	Jinlin city, Jilin Province
10	Fuel alcohol Co., Ltd. (2012)	Jinlin city, Jilin Province
11	Meihekou Wine Co., Ltd.	Meihekou city, Jilin Province
12	Hongyu Biological Feed Co., Ltd.	Gongzuolin city, Jilin Province
13	COFCO Biochemical Energy Co., Ltd.	Gongzuolin city, Jilin Province
14	Jiliang Tianyu biotechnology Co., Ltd.	Changchun city, Jilin Province
15	Sheng Long Alcohol Co., Ltd.	Shuangcheng city, Heilongjiang Province
16	Borun Biotechnology Co., Ltd.	Daqing city, Heilongjiang Province
17	Yangguang corn biochemical Co., Ltd.	Jiamusi city, Heilongjiang Province
18	Sha Qi Industrial Co., Ltd.	Neijiang city, Sichuan Province
19	Jinsheng Yuan bio-chemical Co., Ltd.	Mianzhu city, Sichuan Province
20	Tie Qi Li Shi group	Chengdu city, Sichuan Province
21	Chengde Mountain resort conrporation group Co., Ltd.	Chengde city, Hebei Province
22	Jidong solvent Co., Ltd.	Tiangshan city, Hebei Province
23	Fengyuan Fuel alcohol Co., Ltd.	Bengbu city, Anhui Province
24	Jufeng bio-chemical Co., Ltd.	Liaoyuan city, Liaoning Province
25	Qingdao lucky port Co., Ltd.	Qingdao city, Shandong Province
26	CP Investment (China) Co., Ltd.	Beijing
27	Chifeng Sanli feed Co., Ltd.	Chifeng city, Inner Mongolia
28	Tie Qi Li Shi group	Xian city, Shanxi Province
29	The Scoular company	USA
30	The DELong Co. ZNC	USA

### Birds and housing

This study was approved by the Animal Care and Use Committee of the China Agricultural University. In total, 95 healthy Hy-Line brown roosters (BW = 2.00 ± 0.10 kg, 25 wk of age) were purchased from a local commercial company (Beijing Vocational College of Agriculture Poultry Co., Ltd, Beijing, China). Prior to the feeding trial, every bird was sutured a threaded hollow plastic cap around the vent for screwing a plastic bag to collect excreta [19]. All birds were housed in individual wire cages (47 cm × 60 cm × 36 cm) for an acclimation and preconditioning period for 4 wk. Birds were maintained on a 16-h light schedule and allowed ad libitum access to water. Room temperature was maintained at 23 ± 2°C.

### Experimental procedures

The experiment was conducted according to the modified assay program [20], cockerels were precision fed with 50 g of DDGS after 48 h starvation by Sibbald’s crop intubation [21] method. A randomized incomplete block design was used with 3 periods and 9 replicates totally for each sample. During each period, 90 roosters were randomly allotted to 30 treatment groups, each of groups included 3 birds, three birds were deprived of feed for 48 h to ensure that no feed residues remained in the gastrointestinal tract and then tube fed 50 g of one source of DDGS, and other 5 roosters were fasted throughout to allow for the determination of endogenous energy losses. Excreta was collected for 48 h into a plastic bag and then frozen at -20°C. Before analysis, the samples were lyophilized, allowed to reach equilibrium with the atmospheric moisture for 24 h, weighed and followed by fine grinding (< 2 mm). The birds were returned to a conventional corn-soybean meal diet for 10 d and then rerandomized to treatments for the next replicates. Excreta were collected from 5 fasted roosters in each period to estimate basal endogenous energy.

### Chemical analyses

Chemical composition of DDGS were analyzed according to standard methods [22] for moisture, crude protein, ash, crude fat, Neutral detergent fiber (NDF) and acid detergent fiber (ADF). All DDGS samples and excreta were analyzed for gross energy (GE) with an automatic adiabatic oxygen bomb calorimeter (PARR 1281, PARR Instruments, Moline, IL). AME content of the DDGS samples was determined by the method described by Cozannet [13]. TME were calculated according to the procedure outlined by Gao [23].

### Statistical analyses

The data were analyzed using the one-way analysis of ANOVA of SAS Institute (2003) [24] following a randomized incomplete block design. The individual rooster was the experimental unit. Mean and standard error of the samples were calculated using the PDIFF option of the LSMEANS statement. Correlations were applied to the value of AME, TME, nutrient composition and color data using the CORR procedure of SAS to determine if the value of AME, TME were correlated with nutrient composition and color data (L*, a*, b*). The variance was considered to be significant when *P* < 0.05. Sequential multiple linear regression analysis (stepwise procedure) was employed using nutrient composition (crude protein, ash, crude fat, NDF, ADF), L*, a*, b* and GE as the independent variables and AME or TME as the dependent variable.

## Results

The analyzed crude protein, crude fat, ash, neutral detergent fiber and acid detergent fiber of the 30 DDGS sources were presented in Table 2. Crude fat contents varied from 1.43 to 15.08%, whereas the range of crude protein was narrower (23.30 to 30.61%). Neutral detergent fiber and acid detergent fiber contents ranged from 40.24 to 61.31 and 10.32 to 26.04%, respectively. Ash content ranged from 1.98 to 7.72% in the 30 DDGS samples. Coefficient of determination values was 0.47 (*P* < 0.01) between crude protein and analyzed ADF, and between crude protein and total fat was -0.58 (*P* < 0.01), suggesting there is a high positive correlation between crude protein and ADF, while a negative correlation between crude protein and crude fat content.

**Table 2:** **Chemical composition of the 30 sources of distillers dried grains with solubles on an as-fed basis**^**1**^

**No.**	**Chemical composition of DDGS**				
	**CP**^**2**^	**Fat**^**2**^	**NDF**^**2**^	**ADF**^**2**^	**Ash**
1	26.67	15.08	43.91	12.40	5.11
2	25.64	12.55	50.99	10.32	5.28
3	23.30	4.16	46.08	10.67	7.72
4	23.98	8.42	61.31	19.98	6.07
5	24.45	15.02	55.45	13.31	5.08
6	24.85	14.27	55.45	14.45	5.50
7	28.24	8.07	48.96	17.47	3.93
8	30.31	1.43	58.05	23.60	7.36
9	28.21	10.98	49.59	12.99	4.96
10	29.33	4.73	43.03	16.74	4.18
11	27.99	6.43	48.92	10.53	4.77
12	27.43	10.67	54.94	13.14	4.69
13	28.97	5.65	46.23	15.20	5.00
14	27.68	7.18	43.13	15.99	4.92
15	29.03	4.37	47.36	11.40	4.48
16	23.51	13.81	51.24	13.02	4.95
17	27.04	5.75	48.84	16.45	4.75
18	25.19	12.64	52.53	11.78	5.53
19	27.08	14.21	52.95	14.12	4.59
20	26.83	7.09	40.24	14.85	5.74
21	29.22	2.80	41.82	13.04	4.59
22	30.61	8.05	59.29	24.36	1.98
23	27.90	5.90	58.77	15.32	6.01
24	25.46	12.07	47.11	11.24	4.03
25	27.13	6.89	40.54	13.57	4.50
26	29.27	5.49	43.77	13.59	5.67
27	29.12	7.61	60.02	26.04	3.39
28	24.55	7.41	41.90	15.46	5.66
29	24.66	13.55	57.25	12.27	4.09
30	24.26	12.13	50.30	12.43	4.49
Mean	26.93	8.81	50.00	14.86	4.97
SEM	0.26	0.25	1.49	0.88	0.14

^1^Values presented are from 1 replicate analysis for amino acids and means of duplicate analyses for the other nutrients.

^2^*CP *crude protein, *Fat *crude fat, *NDF *Neutral detergent fiber, *ADF* acid detergent fiber.

DDGS from different provinces of China varied greatly in color, with the Hunterlab L* score ranging from 59.48 (lightest, Source 26) to 30.90 (darkest, Source 2). Color score of a* value varying from 12.39 to 27.71, and b* value varying from 35.27 to 59.75 (Table 3). Hunterlab L*, a*, and b* scores had mean values of 45.68, 19.03 and 46.71. With regard to color score, there was little variation in the a* values among DDGS sources, whereas L* values and b* values were more variable and both highly correlated (0.48, *P* < 0.01) (Table 4).

**Table 3:** **Color characteristics of the 30 sources of distillers dried grains with solubles**^**1**^

**No.**	**Color score**		
	L*	a*	b*
1	39.15	17.27	59.75
2	30.90	27.71	35.27
3	45.58	22.44	36.32
4	51.58	21.51	44.48
5	45.19	18.67	47.32
6	45.69	23.96	46.18
7	38.30	16.31	43.18
8	49.45	16.64	40.11
9	41.79	26.84	47.37
10	42.97	20.60	47.79
11	49.81	20.36	40.64
12	39.61	16.83	37.55
13	40.40	17.90	47.73
14	38.18	18.04	45.81
15	45.27	14.57	39.43
16	36.86	19.67	48.47
17	48.51	17.35	49.57
18	45.90	19.28	51.41
19	46.35	17.63	49.35
20	42.73	17.77	46.02
21	46.52	18.46	49.71
22	57.86	14.75	55.46
23	41.69	24.72	40.94
24	34.69	16.81	42.41
25	58.82	20.17	55.11
26	59.48	17.03	50.28
27	54.28	12.39	48.59
28	40.96	17.04	46.43
29	54.55	19.73	55.26
30	57.28	18.39	53.33
SEM	0.35	0.30	1.11
Mean	45.68	19.03	46.71

^1^Measured by using the Hunterlab color scale; L*, a*, and b* scores are measures of degree of lightness, redness, and yellowness, respectively. Each value is a mean of 6 measurements.

**Table 4:** **Correlation coefficient (r) values between energy and chemical compositions, color score in DDGS samples**^**1,2**^

**Items**	**Items**										
**r**	**GE**	**AME**	**TME**	**NDF**	**ADF**	**fat**	**CP**	**ash**	L*	a*	b*
GE	1.00	0.65^**^	0.65^**^	0.08	-0.07	0.49^**^	0.29	-0.42^*^	-0.18	0.05	0.27
AME		1.00	1.00^**^	-0.15	0.11	0.28	0.28	-0.64^**^	-0.13	-0.28	0.39^*^
TME			1.00	-0.15	0.11	0.28	0.28	-0.64^**^	-0.13	-0.28	0.39^*^
NDF				1.00	0.46^**^	0.27	-0.06	-0.09	0.21	0.04	-0.11
ADF					1.00	-0.33	0.47^**^	-0.23	0.34	-0.43^*^	0.14
EE						1.00	-0.58^**^	-0.22	-0.22	0.21	0.31
CP							1.00	-0.34	0.19	-0.36	0.03
ASH								1.00	-0.17	0.40	-0.44^*^
L*									1.00	-0.29	0.48^**^
a*										1.00	-0.28
b*											1.00

^1^*GE *gross energy, *AME *apparent metabolizable energy, *TME *true metabolizble energy, *NDF *Neutral detergent fiber, *ADF *acid detergent fiber, *CP *crude protein, L*, a*, and b* are measures of degree of lightness, redness, and yellowness, respectively.

^2^Values within a row with * means *P* < 0.05, ** means *P* < 0.01.

The analyzed DM, gross energy (GE), apparent metabolizable energy (AME), true metabolizble energy (TME) contents AME: GE and TME: GE ratio of the 30 DDGS were presented in Table 5. Gross energy content ranged from 16.85 MJ/kg to 22.18 MJ/kg in the 30 DDGS samples and averaged 19.38 MJ/kg. Apparent metabolizable energy ranged from 5.93 to 12.19 MJ/kg and averaged 10.21 MJ/kg. True metabolizable energy ranged from 7.28 to 13.54 MJ/kg and averaged 11.56 MJ/kg. AME: GE ratio in this experiment averaged 52.6% and varied from 35.2 to 69.4%. The TME: GE ratio averaged 59.6% and varied from 43.2 to 77.3% among samples. Coefficient of determination values between b* value and TME was 0.39 (*P* < 0.05), and between crude ash content and TME was -0.64 (*P* < 0.01), suggesting there is a high positive correlation between TME content and b* value, while a negative one between TME content and ash content (Table 4).

**Table 5:** **DM、GE、AME、TME、AME:GE and TME:GE ratio of the 30 sources of distillers dried grains with solubles**^**1,2**^

**No.**	**ME and ME:GE ratio of DDGS**					
	**DM, %**	**GE, MJ/kg**	**AME, MJ/kg**	**TME, MJ/kg**	**A/G, %**	**T/G, %**
1	93.14	21.02	10.71	12.06	50.96	57.39
2	92.65	20.25	10.60	11.95	52.35	59.03
3	91.77	16.85	5.93	7.28	35.21	43.23
4	93.18	19.13	7.11	8.46	37.18	44.24
5	92.18	20.46	11.16	12.51	54.52	61.12
6	93.02	20.80	11.09	12.44	53.31	59.81
7	91.85	20.92	11.38	12.73	54.40	60.86
8	93.08	16.93	6.94	8.29	40.96	48.95
9	92.39	20.08	10.30	11.65	51.29	58.02
10	91.05	19.95	11.43	12.79	57.31	64.09
11	91.30	18.57	9.04	10.39	48.68	55.96
12	92.53	19.76	10.44	11.80	52.86	59.70
13	90.18	18.50	10.46	11.81	56.52	63.83
14	89.33	19.65	12.06	13.42	61.40	68.28
15	91.21	18.64	8.63	9.99	46.33	53.58
16	90.99	17.29	8.52	9.87	49.27	57.09
17	90.01	19.65	12.19	13.54	62.04	68.91
18	92.08	20.31	10.79	12.14	53.13	59.79
19	93.51	20.82	10.77	12.12	51.73	58.22
20	89.30	19.48	11.14	12.50	57.21	64.14
21	90.26	19.04	11.05	12.40	58.02	65.12
22	94.60	22.18	11.86	13.21	53.45	59.54
23	93.07	19.09	8.99	10.35	47.12	54.20
24	91.60	19.83	11.00	12.35	55.48	62.29
25	89.04	16.93	8.31	9.66	49.07	57.06
26	89.58	19.42	10.51	11.86	54.13	61.09
27	93.10	17.03	11.81	13.16	69.36	77.29
28	87.91	19.52	11.18	12.53	57.26	64.18
29	90.90	19.51	10.35	11.71	53.08	60.01
30	91.05	19.65	10.47	11.82	53.27	60.15
Mean	91.53	19.38	10.21	11.56	52.56	59.57
SEM	0.07	0.11	0.30	0.30	0.02	0.02

^1^Values represent a single analysis of each DDGS sample.

^2^*DM *dry matter, *GE *gross energy, *AME *apparent metabolizable energy, *TME *true metabolizable energy, *A/G *AME: GE ratio, *T/G *TME: GE ratio.

Equations with which to estimate the ME on the basis of GE, color scores and chemical composition were developed based on 1 to 6 variables (Table 6). The best single indicator of ME was GE (R^2^ = 0.42). The other variables (ash, NDF, ADF, crude fat, L*) improved the accuracy of the ME prediction equations (R^2^ = 0.58, 0.63, 0.67, 0.69, 0.70). Regression equation of the content of AME was AME = 6.57111 + 0.51475 GE - 0.10003 NDF + 0.13380 ADF + 0.07057 fat - 0.57029 Ash - 0.02437 L. Regression equation of the content of TME was TME = 7.92283 + 0.51475 GE - 0.10003 NDF + 0.13380 ADF + 0.07057 fat - 0.57029 Ash - 0.02437 L.

**Table 6:** Prediction equations for ME of DDGS based on chemical composition and color score

**n**	**Prediction equations of DDGS**		
	**Variable**^**1**^	**Prediction equation**	**R**^**2**^
1	GE	AME = -4.69002 + 0.76890 GE	0.42
		TME = -3.33829 + 0.76890 GE	
2	GE, ash	AME = 2.72386 + 0.55076 GE - 0.64175 ash	0.58
		TME = 4.07558 + 0.55076 GE - 0.64175 ash	
3	GE,NDF, ash	AME = 5.42832 + 0.56455GE - 0.05729 NDF - 0.66330 ash	0.63
		TME = 6.78004 + 0.56455GE - 0.05729NDF - 0.66330 ash	
4	GE,NDF, ADF, ash	AME = 3.86442 + 0.62133 GE - 0.08157 NDF + 0.08383 ADF - 0.57630 ash	0.67
		TME = 5.21614 + 0.62133 GE - 0.08157 NDF + 0.08383 ADF - 0.57630 ash	
5	GE,NDF, ADF, ash, fat	AME = 5.03286 + 0.54081 GE - 0.10615 NDF + 0.12914 ADF + 0.08000 fat - 0.52751 ash	0.69
		TME = 6.38458 + 0.54081 GE - 0.10615 NDF + 0.12914 ADF + 0.08000 fat - 0.52751 ash	
6	GE,NDF, ADF, ash, fat, L	AME = 6.57111 + 0.51475 GE - 0.10003 NDF + 0.13380 ADF + 0.07057 fat - 0.57029 ash - 0.02437 L	0.70
		TME = 7.92283 + 0.51475 GE - 0.10003 NDF + 0.13380 ADF + 0.07057 fat - 0.57029 ash - 0.02437 L	

^1^*GE *gross energy, *NDF *Neutral detergent fiber, *ADF *acid detergent fiber, *fat *crude fat, *L *luminance.

## Discussion

This experiment demonstrated that the degree of yellowness and the ash content of the corn DDGS highly correlated with the content of TME. This correlation seemed to be particularly exacerbated for the degree of lightness, which had the greatest variability among the 30 DDGS sources evaluated. These results implied that colorimetric measurements, such as L* and b* and chemical composition may provide a rapid method for identifying DDGS sources with good or poor energy availability.

### Chemical composition of corn DDGS

The average composition of the corn DDGS used in the present trials is in agreement with previous literature data [10,12,18] and different with wheat DDGS, because wheat DDGS is typically higher in protein (CP: 32% - 39%, DM) and considerably lower in fat (fat: 3.6% - 5.6%, DM) than corn DDGS [25-29]. In our trials and in connection with the high number of samples obtained from China plants, an important variability in chemical composition was observed among samples, probably related to the characteristics of the grains and the process used to produce ethanol [10,30,31]. However, no quantitative and even qualitative information on the processing technologies for the DDGS batches that were studied in the present trials was available. Therefore, we could not relate the nutritional values to the procedures but only to the chemical and physical characteristics of the DDGS. The range in chemical composition of DDGS was fully expected because the chemical composition of DDGS can be influenced by the degree of starch fermentation, heat processing, proportion of solubles added back to the distillers dried grains, and drying method at a particular production facility [32,33].

Furthermore, the color attributes (L, a and b values) did not show good correlations with compositional traits. However, b values correlated negatively with ash. This observation indicates that some pre-fractionation procedures (such as fiber and impurities removal) could change color attributes, they could lead to lower ME content in DDGS.

### Color scores of corn DDGS

It is clear from previous studies that during conversion of corn to ethanol, although the principle is similar, there is a great variation in grain material and methods used among processing plants [34,35]. Still others use different parameters (pH, temperature, duration, sources of enzyme, type of equipment, size of screens used for grinding, etc.). Thus, the variations in grain and methods used among plants, plus complex interactions of many factors during the process within a plant, would lead to great variations in color score in the original DDGS samples from different plants. Previous observations showed that the range of Hunterlab L*, a* and b* scores were from 28.0-62.9 (L*), 4.1-14.47 (a*) and 5.3-46.3 (b*) [10,15,17,18,36,37]. The DDGS samples evaluated by this experiment had L* values that ranged from 30.90 to 59.48, which agrees with previous observations. However, the values of a* (12.39 -27.71) and b* (35.27 to 59.75) varied greater than previous researches. The results indicated that the color score of DDGS from China has a higher vary than the samples from America. Furthermore, among color attributes, L* and b* values had a very good positive correlation.

### Availability of energy

It is clear from this study that the content of AME and TME of DDGS sources (sample no. 29 and 30) from America was similar to the values reported by the National Research Council (NRC 1994) [38] for DDGS on 93% DM basis. However, the AME and TME values of DDGS samples from China (sample 3, 4, 8, 11, 15, 16, 23, 25) were lower than that reported in the NRC (1994) [38]. One possible reason for the inconsistency is that the raw materials and the processing methods are different between two countries [8]. In fact, our study indicates that DDGS with high a^*^ value and low L^*^ value would contribute to the lower ME among DDGS samples. Similar observations have been done by Cromwell et al. [10] and Fastinger et al. [17] for DDGS when fed to poultry. This phenomenon affects mainly low starch content. DDGS and is likely associated with Maillard reaction occurrence producing brown compounds and a lower availability of amino acids [17] and energy (present trials). Unfortunately, in our trials, the samples with high redness values also had the highest ADF content (Table 2). This also means that the conventional analyses for feed evaluation are insufficient for characterizing that have been overheated.

### Energy digestibility of DDGS

Energy digestibility in poultry depends on genetic effects [39] and the bird BW or its degree of development. A high correlation between redness and ME: GE ratio suggested the energy digestibility of DDGS could be predicted by the color score. However, there is insufficiently documented about the relationship between ME: GE ratio and color value. In conclusion, our study provided the original data and more researches should be confirmed.

### Prediction energy equation of DDGS

The use of composition analysis to predict energy values of feed ingredients for poultry is not novel [40-42]. Recently, prediction equations derived from composition analysis have been developed for meat and bone meal [43], wheat DDGS [44] and corn DDGS [18]. Batal and Dale [18] reported that the best predictors of TME for DDGS were fat, fiber, protein, and ash (R^2^ = 0.45). Using a covariate model and simple linear regression, Cozannet et al. [13] determined that the AME of wheat DDGS could be predicted with luminance (R^2^ = 0.77) and ADF (R^2^ = 0.79). In this study, however, ash had the strongest correlation with TME rather than NDF or ADF. This could be a result of using wheat DDGS rather than corn DDGS. Furthermore, previous research developed TME prediction equations using only the DDGS from the U.S. [18]. As a result, variation in chemical composition between samples was not as large as that observed between the diverse arrays of corn DDGS used in the current study. For example, the crude ash content of samples used by Batal and Dale [18] ranged from 3.9 to 5.4%, whereas crude ash in the current study ranged from 1.98 to 7.72%, respectively.

## Conclusions

In conclusion, the quality of corn DDGS from this research varied greater than the corn DDGS published from previous observations. Metabolizable energy content had a relation with chemical composition and color score. This experiment suggested that measuring the chemical composition and color score of a corn DDGS sample may provide a quality parameter for identifying corn DDGS sources energy digestibility and metabolizable energy content. The correlationship between ME content and chemical composition and color score of DDGS from wheat, soghum or other blend grains needed further study.

## Competing interests

The authors declare that they have no competing interests.

## Authors’ contributions

YZJ carried out the statistical analysis and drafted the manuscript. ZLH participated in the chemical analysis. MQG, JYZ and JC conceived the study, participated in its design and coordination, and helped draft the manuscript. All authors read and approved the final manuscript.
